# Impact of Obesity on Atrial Electrophysiological Substrate

**DOI:** 10.3390/jcdd10080342

**Published:** 2023-08-08

**Authors:** Corina Schram Serban, Natasja M. S. de Groot

**Affiliations:** 1 Department of Cardiology, Erasmus University Medical Center, 3015 GD Rotterdam, The Netherlands; corinaserban31@gmail.com; 2 Department of Microelectronics, Circuits and Systems, Faculty of Electrical Engineering, Mathematics and Computer Sciences, Delft University of Technology, 2628 CD Delft, The Netherlands

**Keywords:** obesity, atrial fibrillation, electrophysiology

## Abstract

(1) Background. Obesity is a well-established worldwide recognised risk factor for atrial fibrillation (AF). Prior review papers reported on the associations between obesity and AF development, but not on the relation between obesity and atrial electrophysiology. We therefore conducted a systematic review to describe the current knowledge of the characteristics of the atrial electrophysiological substrate in obese individuals and how they relate to the development of AF. (2) Methods. A search was conducted in Pubmed, Embase, and the Cochrane Library for publications evaluating the impact of obesity on atrial electrophysiology, electrical substrates, and their relation to the development of AF. (3) Results. A systematic literature search retrieved 477 potential publications based on the inclusion criteria; 76 full-text articles were selected for the present systematic review. The literature demonstrated that obesity predisposes to not only a higher AF incidence but also to more extensive atrial electrophysiological abnormalities increasing susceptibility to AF development. (4) Conclusion. Obesity may predispose to an overall increase in atrial electropathology, consisting of an increase in the slowing of the conduction, conduction block, low-voltage areas, and complex fractionated electrograms. To determine the impact of obesity-induced atrial electrical abnormalities on the long-term clinical outcome, further prospective studies are mandatory.

## 1. Introduction

**Figure 1:**
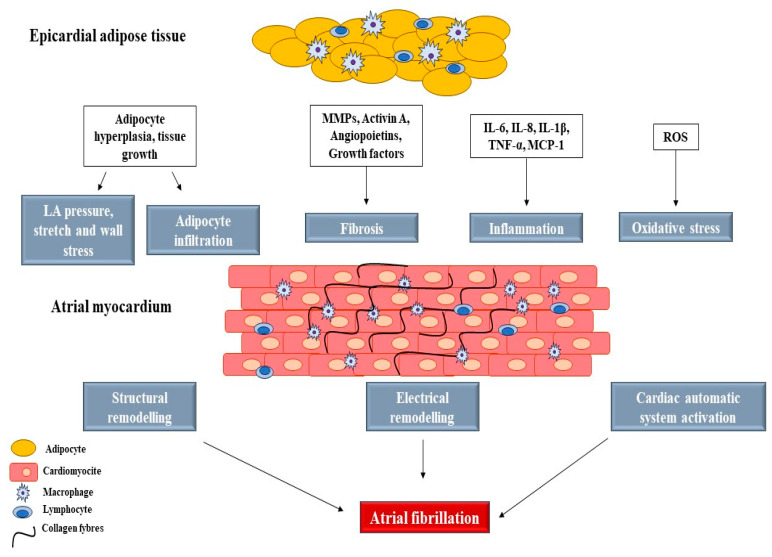
Role of epicardial fat in the development of AF. MMP, matrix metalloproteinase; TNF, tumour necrosis factor; MCP, monocyte chemoattractant protein; ROS, reactive oxygen species.

Obesity is a well-established worldwide recognised risk factor for atrial fibrillation (AF) [1,2,3,4]. The link between obesity and the development of AF includes common risk factors (e.g., metabolic syndrome [4,5] and coronary artery disease [6]), as well as atrial substrate modifiers (e.g., epicardial adipose tissue (EAT) [7,8,9,10,11,12,13,14,15,16,17], hypoxia [18,19], and fibrotic remodelling of the subepicardium [20,21])). The proarrhythmogenic substrate of obesity is associated with left atrial (LA) size [10,22,23,24,25], increased inflammation, oxidative stress through elevated reactive oxygen species (ROS), and autonomic dysregulation mediated by increased adipocytokines and proinflammatory cytokines [19]. EAT is an endocrine organ and a source of proinflammatory cytokines including TNF-α, IL-1, IL-6, the monocyte chemoattractant protein-1 (MCP-1), and profibrotic factors such as transforming growth factors (TGFs) and matrix metalloproteinases (MMPs) acting in a paracrine way on the myocardium [26]. Figure 1 shows how these molecules diffused in the pericardial sac contribute to the structural remodelling of the atria [26].

The increased body mass index (BMI) was associated with atrial electrophysiological modifications including conduction [6,27,28,29,30,31,32,33,34,35,36,37] and potential voltage abnormalities [29,30,38,39,40], as well as the presence of fractionated electrograms [15,41,42,43]. Furthermore, obesity was linked to the development of a new onset [44,45,46,47,48,49], post-operative AF [6,38,44,50,51,52,53,54], with influence on AF ablation procedure outcomes [55,56,57,58,59,60,61,62,63,64,65,66].

The association between obesity and AF development has been presented in several review papers [1,2,3,5,16,19,21,46,51,66,67,68,69,70,71] in the past; however, a literature review reporting on obesity-related atrial electrophysiological abnormalities and their contribution to AF development is still missing. We therefore conducted a systematic review to describe the current knowledge regarding the characteristics of atrial electrophysiology in obese individuals and how they relate to development of AF.

## 2. Materials and Methods

The reporting of this systematic review was guided by the standards of the Preferred Reporting Items for Systematic Review and Meta-Analysis (PRISMA) Statement [72]. The review was also submitted for registration in the PROSPERO International prospective register of systematic reviews (ID: 444962). The search was conducted in PubMed, Embase, and Medline Library for publications evaluating the impact of obesity (BMI) on atrial electrophysiology and the electrical substrate and how it relates to the development of AF. The search terms included: “Heart Conduction System” or “atrial conduction abnormality” or “atrial conduction abnormal” OR “conduction abnormalities” or “low voltage area” or “low voltage” or “complex fractionated potential” or “fractionat” or “fragment” or “CFAE” or “CFE” or “AF nest” or “CEA” or “atrial mapping” or “conduction delay” or “conduction block” or “Heart Atria” or “Interatrial Block” or “Heart Block” or “Epicardial Mapping” or “Electrophysiology” or “Cardiac Electrophysiology” or “Catheter Ablation” AND “Atrial Fibrillation” AND “Interatrial Block” AND “Heart Block” AND “Epicardial Mapping” AND “Electrophysiology” or “Cardiac Electrophysiology” AND “Catheter Ablation” AND “Obesity” AND “Adipose Tissue” AND “Adipocytes”.

No language restriction was imposed. In addition, a manual search through the reference list was performed for original and review publications.

### 2.1. Inclusion Criteria

Eligible studies met the following criteria:

Human subjects

Animal subjects

Clear definition of normal and elevated BMI (BMI ≥ 25 kg/m^2^)

Atrial endocardial and epicardial mapping procedures

Atrial electrophysiological abnormalities in obese patients

Atrial fibrillation pre/post-operative

AF burden in obese patients

Catheter ablation for AF

Publications failing to meet the above-mentioned criteria were excluded from the review.

### 2.2. Study Selection

All titles were independently screened by two investigators, including the titles and abstracts of the identified articles. Relevant full-text publications were investigated to determine their eligibility. A consensual agreement was reached.

### 2.3. Quality Assessment

Study quality was assessed following the key study design components recommended by the MOOSE Group [72]. The key points included: (1) the study was prospective; (2) clear inclusion and exclusion description criteria; (3) study sample was representative of the population; (4) clear definition of elevated BMI; (4) clear description of mapping procedure; (5) clear description of electrophysiological abnormalities and how they relate to BMI; (6) description of the type of AF and moment of occurrence; (7) clear definition of outcomes and outcome assessment; and (8) proper adjustment for confounding factors for elevated versus normal BMI categories for AF occurrence. One point was attributed to each article that contained at least one key point. The articles that contained less than 8 key points were considered to be suboptimal due to the potential underestimation of the reported characteristics.

## 3. Results

**Figure 2:**
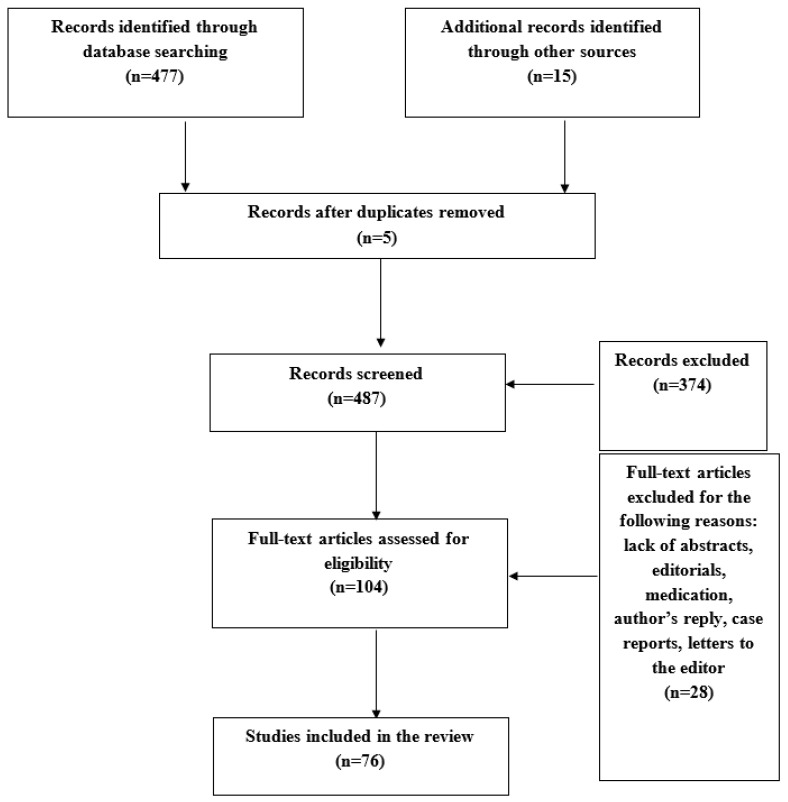
PRISMA flow chart depicting literature selection. n = number of articles.

The systematic literature search retrieved 477 potential publications for inclusion. Figure 2 shows a flow chart depicting the study selection procedure. Following thorough screening of the titles and abstracts of the potentially relevant literature, 351 articles were excluded based on relevance. The literature lacking abstracts, editorials, letters to the editor, case reports, and duplicates were further excluded from the study (N = 28). Finally, the literature describing the medication trials and the presence of obstructive sleep apnoea were also excluded (N = 25). The remaining 76 articles were included in the systematic review. The relevant literature for atrial electrophysiological abnormalities, including conduction and voltage abnormalities, as well as presence of complex fractionated electrograms, is depicted in Table 1.

### 3.1. Obesity and Conduction Abnormalities

Atrial conduction abnormalities are by far the most investigated electrophysiological characteristic related to elevated BMI [27,28,29,30,31,32,33,34,35,36,48,73,74,75]. Conduction abnormalities were investigated by evaluating the P wave duration and morphology [33,34,73,74,75,76]. Friedman et al. investigated the relation between the presence of epicardial fat and atrial conduction describing P wave indices (PWI) in a community-based (N = 1946; 45% female) population. These PWI included PR interval, P wave duration, P wave amplitude, P wave area, and P wave terminal force. The main outcome of this study showed that pericardial fat was associated with atrial conduction as measured by the PWI [73]. P wave duration and P wave terminal force were significantly correlated with pericardial fat, whilst PR interval was associated with BMI. Correlations were, however, mild or moderate in magnitude [73]. As part of the Atherosclerosis Risk in Communities (ARIC) Study, Magnani et al. investigated the impact of obesity and metabolic syndrome on electrophysiological properties by analysing the alteration of P wave indices in the resting 12-lead ECG. Secondly, they sought to distinguish the racial differences of the impact of obesity and metabolic syndrome on P wave indices [74]. The study showed that higher BMI was associated with progressively longer PR intervals, P wave maximum durations, and larger P wave terminal forces. These findings remained significant after adjusting for demographic, clinical, and metabolic covariates [74]. Similar findings were previously presented by Babcock et al. in the Multicentric Study of Atherosclerosis (MESA) which reported that increased BMI was also associated with the prolongation of P wave indices after adjusting for age, gender, ethnicity, and pericardial fat [76]. However, the MESA study’s investigation of BMI and P wave indices did not adjust for clinical variables [74] compared to the ARIC study. Furthermore, Vaidean et al. demonstrated that a 5-unit increase in BMI lead to a progressive increase in ECG indices, including increased P wave duration by 1.9 ms (95% CI 1.5–2.2) and PR interval by 2.4 ms (95% CI 1.9–3.0) [33].

P wave prolongation and electromechanical delay were also investigated in children with obesity by Temiz et al. [33]. Compared to healthy controls, time interval from the onset of the P wave on the surface ECG to the start of the late diastolic wave interval septal, in addition to the inter- and intra-atrial electromechanical delays assessed by tissue Doppler echocardiography, which were prolonged in obese patients [33].

The impact of obesity on atrial conduction abnormalities has also been investigated by utilizing endocardial mapping procedures [28,29,30,31] in either human [29,31] or animal studies [28,30].

Munger et al. investigated electrophysiological and hemodynamic features in 63 (44 obese and 19 nonobese) patients enrolled for catheter-based radiofrequency pulmonary vein isolation procedure for drug-refractory AF [31]. Endocardial mapping showed that obese patients with AF had slower conduction from the left atrium (LA) into the pulmonary vein [31]. Moreover, obese patients had a shorter effective refractory period in the LA and proximal and distal pulmonary veins compared to normal BMI patients [31]. Similar findings on the slowing of the conduction were presented by Mahajan et al. in a group of 26 (16 obese and 10 nonobese) patients undergoing electroanatomical mapping of the LA prior to AF ablation therapy [29]. In this patient group, obesity was associated with global reduction in conduction velocity in the LA [29]. Moreover, obesity was associated with increase in all measures of EAT, with predominant distribution adjacent to the posterior LA and the atrioventricular groove [29]. EAT was also better correlated with conduction velocity in the LA compared to BMI [29]. The authors therefore concluded that obesity is associated with electroanatomical remodelling of the atria, with the electrophysiological changes being more pronounced in regions adjacent to the epicardial fat depots, which suggested a role of fat depots in the development of the AF substrate.

Animal studies of high-fat diet-induced obesity also provided insights into the relationship between conduction abnormalities and obesity [28,30]. Mahajan et al. investigated the development of AF-related structural and electrophysiological substrates in a calorie-dense diet-fed sheep model. The outcomes of this study showed that chronically obese sheep had more total body fat, larger LA volume, and higher LA and pulmonary artery pressure, in addition to reduced atrial conduction velocity with increased conduction heterogeneity [30]. Obesity was also associated with increased and prolonged AF episodes; hence, a larger cumulative duration of AF episodes [30]. Alternatively, in a high-fat diet porcine model, Okumura et al. found that despite shorter effective refractory periods in the pulmonary veins, a high fat diet induced no conduction disturbance in the atria. However, the sheep enrolled in the study by Mahajan et al. were fed a high calorie diet for 32 weeks, compared to only 18 weeks for the pigs presented by Okumura et al.

Epicardial mapping procedures have also been applied for the characterisation of the atrial substrate in obese human [6,32] and animal models [27,36,48]. Schram-Serban et al. investigated the difference in severity and extensiveness of conduction disorders in obese (N = 106) patients, which was compared to nonobese (N = 106) patients and measured at a high-resolution scale. This study showed that the overall incidence in the conduction delay, conduction block, and continuous areas of conduction delay or block was higher in obese individuals, compared to patients with normal BMI [6]. These findings were particularly significant in Bachmann’s bundle and the pulmonary vein areas. In addition, obese patients had a higher incidence of early, de novo post-operative AF [6]. Though BMI was an independent predictor for the development of post-operative AF, the multivariate analysis showed that the associated risk factors, include hypertension, hyperlipidaemia, and left atrial enlargement, were also independently associated with AF occurrence [6].

Similar findings were observed by Nallilah et al. in a study investigating the role of EAT on the atrial electrical substrate underlying AF in 19 patients who enrolled for coronary artery bypass grafting surgery and in whom right atrial high density epicardial mapping was performed [32]. A higher local EAT volume correlated with slowed atrial conduction and conduction heterogeneity [32].

In the high calorie fed sheep model, Abed et al. observed a decrease in conduction velocity, and an increase in conduction heterogeneity, AF inducibility, and episodes [48].

### 3.2. Obesity and Low-Voltage Areas

Low-potential voltages are most commonly considered a marker for atrial fibrosis [77]. Fibrosis is a known risk factor for AF development [78] and is also more pronounced in patients with AF [78].

Low-voltage areas in the atria have been described in obese patients [29,38,39], obesity animal models [30], or in experimental studies by using whole-cell patch clamp techniques [36,40]. In 16 obese compared to nonobese patients, endocardial mapping of the LA during sinus rhythm prior to AF ablation therapy revealed significantly lower mean potential voltages in the posterior LA walls in obese patients. In the LA, the incidence of low-voltage potentials was significantly higher in the obese (13.9%) compared to the nonobese patients (3.4%) [29].

A more comprehensive approach for defining the incidence and extent of low-voltage atrial areas in obese patients is presented by Schram-Serban et al. during high-resolution epicardial mapping of 212 patients (106 obese vs. 106 nonobese) undergoing cardiac surgery [38]. The overall incidence of low-voltage potentials was significantly higher in obese compared to nonobese patients with a predilection for the LA and BB [38]. Low-voltage areas were predominantly found at BB [38]. Moreover, this study also showed that both BMI and the percentage of low-voltage areas were independently associated with the development of early post-operative AF as well as the presence of hypertension and left atrial enlargement [38].

The influence of obesity on the occurrence of atrial low-voltage areas has also been demonstrated in animal models [30]. Endocardial electroanatomical mapping studies during sinus rhythm, demonstrated in a calorie-dense diet fed ovine models a significant reduction in potential voltages in the posterior LA area [30]. Moreover, this study also showed an increase in regional voltage heterogeneity in the obese animals, as compared to the control animals [30].

### 3.3. Obesity and Atrial Complex Fractionated Electrograms

Complex fractionated electrograms (CFAEs) are defined as, e.g., continuous atrial electrogram activity, complex fractionated potentials, or simply atrial electrograms with short mean cycle lengths [79]. CFAEs are believed to indicate areas of conduction slowing or blocks, anchor points for continuous re-entry, locations bordering high-frequency sites where fibrillatory conduction and wave breaks occur, overlap or multiple activation waves, or sites of autonomic innervation [79]. Identification of CFAEs therefore provides supplemental information on the extent of electrical remodelling. A clear description of the extensiveness of CFAEs in obese individuals in the current literature is still limited [28,29,30,42].

When performing electroanatomical mapping of the LA during sinus rhythm in patients with AF, more complex fractionated potentials and double potentials were found in obese patients compared with nonobese patients [29]. CFAE were also stronger correlated with LA EAT than BMI [29].

Also, in the obesity sheep model, electroanatomical mapping of both atria performed during sinus rhythm showed that the LA contained more fractionated and long double potentials compared to the right atrium in both the obese and nonobese groups [30]. However, the incidence of double potential and/or fractionated potentials was significantly higher in obese compared to nonobese sheep [30]. Alternatively, Okumura et al. constructed CFAE maps during induced-AF of both atria in a small group of 10 (5 obese vs. 5 nonobese) pigs. These maps showed that the spatial distribution of CFAE was similar in both groups [28]. However, induced-AF was only sustained in three out of five pigs in the nonobese group.

Kanazawa et al. also investigated the link between pericardial fat and CFAEs in the development of AF. Pericardial fat volume was assessed using CT imaging and CFAE regions were mapped during AF using three-dimensional endocardial mapping of the LA [42]. Pericardial fat was most pronounced at the roof, interatrial septum, and the posterior LA wall. The pericardial fat areas at the roof and anterior LA were significantly larger than at the lateral, septal, and posterior LA [42]. The author therefore concluded that the regional distribution of pericardial fat volume was similar to that of CFAE areas, resulting in a significant correlation between the total LA pericardial fat volume and total CFAE area, particularly in the LA roof area [42].

### 3.4. Obesity, Conduction Abnormalities, and Development of Early Post-Operative AF (EPOAF)

Previous studies have demonstrated that BMI, a measure of overall adiposity, is a strong, independently associated factor with not only AF but also PoAF [31,53,80,81,82]. In a meta-analysis by Phan et al., the association between obesity and PoAF in patients without previous history of AF showed that obesity was associated with a significant risk of PoAF. Furthermore, Munger et al. found that obesity was associated with a shorter effective refractory period (ERP) in the LA, proximal, and distal PV in 63 patients with AF undergoing catheter ablation. In our study, we found that obese patients had a higher incidence of EPOAF. Multivariate analysis showed that a 1.064 unit increase in BMI resulted in a higher incidence of EPOAF (*p* = 0.037). Previous studies demonstrated that a 1-unit rise in BMI increases the frequency of newly developed AF by 4% [83]. Comorbidities contributing to the development of EPOAF also include the incidence of MVD, hypertension, and left atrial enlargement [84]. By using high-resolution atrial epicardial mapping during SR, Schram-Serban et al. demonstrated that the incidence of the conduction block in the entire atria independently predicted the development of EPOAF. These findings suggest that obesity-related heterogeneity in conduction plays an important role in the development of EPOAF [6].

### 3.5. Obesity, Voltage Abnormalities and Development of Early Post-Operative AF (EPOAF)

Atrial fibrosis is a feature in obesity-related structural remodelling. Prior studies demonstrated relations between the histological evidence of increased atrial fibrosis and the indirect evidence of reduced endocardial atrial potential voltages, though this relationship remains controversial [85,86]. Obesity is associated with hypoxia of the expanding adipose tissue resulting in adipose tissue fibrosis and production of various adipo-cytokines, including the TGFβ family [87]. The combination of increased epicardial adiposity, atrial fibrosis, and altered three-dimensional atrial architecture could be pro-fibrillatory with an increased likelihood of conduction heterogeneity that may play a role in initiation and perpetuation of atrial tachyarrhythmias such as AF [85]. Schram-Serban et al. performed high resolution and density mapping of the atria and found that the percentage of unipolar low-voltage potentials was independently associated with the development of early post-operative AF. The author would not abbreviate this in obese patients [38]. The study also showed a correlation between the incidence of unipolar low-voltage potentials and the percentage of the conduction block in the entire atria [38], suggesting that the correlation between these two electrophysiological parameters could explain the higher risk of early post-operative AF development in obese patients.

### 3.6. Reversibility of Obesity-Induced Electropathology

Experimental studies have shown that atrial electropathology is reversible with weight loss [88]. In an obesity sheep model, Mahajan et al. demonstrated that a 30% reduction in weight resulted in a significantly lower total body fat, LA pressure, inflammation, endothelin-B receptor expression, and atrial fibrosis [88]. Consequently, an increased effective refractory period, improved conduction velocity, decreased conduction heterogeneity, and reduced AF inducibility were observed [88].

## 4. Conclusions

Obesity may predispose to a higher incidence and extensiveness of atrial electrophysiological abnormalities, increasing the risk for the development of AF. Obesity-related atrial electropathology consists of an increase in the slowing of the conduction, conduction block, low-voltage areas, and complex fractionated electrograms. Further prospective studies are mandatory [6] to (1) determine the impact of obesity-induced atrial electrical abnormalities on the long-term clinical outcome and (2) investigate whether obesity-related atrial electropathology is reversible with weight management [88].

## Figures and Tables

**Table 1:** Literature overview describing atrial electrophysiological abnormalities.

Atrial Electrophysiological Abnormalities	Author	Title	Type of Study	Research Methods
Conduction abnormalities	Schram-Serban, C., et al. [6]	Heterogeneity in Conduction Underlies Obesity-Related Atrial Fibrillation Vulnerability	Human	Epicardial high resolution mapping. In obese patients, the overall incidence of CD (3.1% vs. 2.6%; *p* = 0.002), CB (1.8% vs. 1.2%; *p* < 0.001), and cCDCB (2.6% versus 1.9%; *p* < 0.001) was higher and CD (*p* = 0.012) and cCDCB (*p* < 0.001) lines were longer.
	Hohl, M., et al. [27]	Concomitant Obesity and Metabolic Syndrome Add to the Atrial Arrhythmogenic Phenotype in Male Hypertensive Rats	Experimental (Muridae)	Telemetric monitoring (implantation of telemetric sensors) Increase in total atrial activation time and percentage of regions with slow conduction during rapid pacing. Longer-induced AF episodes in the obese group.
	Okumura, Y., et al. [28]	Effects of a high-fat diet on the electrical properties of porcine atria	Experimental (Suidae)	Endocardial mapping. ERPs of the pulmonary vein were shorter (*p* < 0.05), and AF lasted longer in the high fat diet group than in the control group (80 (45–1350) vs. 22 (3–30) s, *p* = 0.0212).
	Mahajan, R., et al. [30]	Electroanatomical Remodeling of the Atria in Obesity: Impact of Adjacent Epicardial Fat	Human	Endocardial mapping Obesity was associated with reduced global conduction velocity (0.86 ± 0.31 m/s vs. 1.26 ± 0.29 m/s; *p* < 0.001), increased fractionation (54 ± 17% vs. 25 ± 10%; *p* < 0.001), and regional alteration in voltage (*p* < 0.001). Greater voltage heterogeneity (*p* = 0.001) and increased low-voltage areas (13.9% vs. 3.4%; *p* < 0.001) in the obese group.
	Mahajan, R., et al. [29]	Electrophysiological, Electroanatomical, and Structural Remodeling of the Atria as Consequences of Sustained Obesity	Experimental (Ovis)	Endocardial mapping Chronically obese sheep demonstrated increased conduction heterogeneity (*p* < 0.001); increased fractionated electrograms (*p* < 0.001); decreased posterior LA voltage (*p* < 0.001); and increased voltage heterogeneity (*p* < 0.001). Obesity was associated with more episodes (*p* = 0.02), prolongation (*p* = 0.01), and greater cumulative duration (*p* = 0.02) of AF.
	Munger, T.M., et al. [31]	Electrophysiological and hemodynamic characteristics associated with obesity in patients with atrial fibrillation	Human	Endocardial mapping At a 600 ms pacing cycle length, obese patients had a shorter effective refractory period (ERP) in the left atrium (251 ± 25 ms vs. 233 ± 32 ms, *p* = 0.04), and in the proximal (207 ± 33 ms vs. 248 ± 34 ms, *p* < 0.001) and distal (193 ± 33 ms vs. 248 ± 44 ms, *p* < 0.001) PV than normal BMI patients.
	Nalliah, C.J., et al. [32]	Epicardial Adipose Tissue Accumulation Confers Atrial Conduction Abnormality	Human	High-density epicardial electrophysiological mapping Higher local epicardial adipose tissue volume clinically correlated with slowed conduction, greater electrogram fractionation, and increased fibrosis. Cardiomyocyte culture studies using multielectrode arrays showed that cardiac adipose tissue-secreted factors slowed conduction velocity.
	Temiz, F., et al. [33]	Evaluation of Atrial Electromechanical Delay in Children with Obesity	Human	Transthoracic echo and tissue Doppler echocardiography. Obese patients had significantly lengthened P wave on surface ECG to the beginning of the late diastolic wave (PA) lateral, PA septum, and intra- and inter-atrial electromechanical delays when compared with the control group (*p* < 0.001, *p* = 0.001, *p* < 0.001, and *p* < 0.001, respectively).
	Vaidean, G.D., et al. [34]	Atrial electrocardiography in obesity and hypertension: Clinical insights from the Polish-Norwegian Study (PONS).	Human	Digital standard 12-lead resting ECG. Each 5-unit increment in the body mass index (BMI) increased P wave duration by 1.9 ms (95% CI 1.5–2.2) and PR interval by 2.4 ms (95% CI 1.9–3.0), with similar trends for central obesity, even among those without obesity by BMI.
	Lin, Y.K., et al. [36]	Adipocytes modulate the electrophysiology of atrial myocytes: implications in obesity-induced atrial fibrillation	Experimental (Leporidae)	Whole-cell patch clamp. Compared to the control LA myocytes (n = 22), LA myocytes incubated with epicardial (n = 17), retrosternal (n = 18), or abdominal adipocytes (n = 22) had longer (80 ± 3, 109 ± 6, 109 ± 6, and 110 ± 7 ms, *p* < 0.001) 90% AP durations (APD(90)). Epicardial adipocyte-incubated LA myocytes had larger late sodium currents, L-type calcium currents, and transient outward potassium currents, but smaller delayed rectifier potassium and inward rectifier potassium currents than the control LA myocytes.
	Friedman, D.J., et al. [73]	Pericardial fat is associated with atrial conduction: the Framingham Heart Study	Human	Standard 12-lead resting ECG. Each 1-SD increase in pericardial fat was significantly associated with PR interval (β = 1.7 ms, *p* = 0.049), P-duration (β = 2.3 ms, *p* < 0.001), and P-terminal (β = 297 μV·ms, *p* < 0.001) among women; and P-duration (β = 1.2 ms, *p* = 0.002), P-amplitude (β = -2.5 μV, *p* < 0. 001), and P-terminal (β = 160 μV·ms, *p* = 0.002) among men.
	Magnani, J.W., et al. [74]	P wave indices, obesity, and the metabolic syndrome: the atherosclerosis risk in communities study	Human	Standard 12-lead resting ECG. In multivariable analyses, there was significant, progressive increases in PR interval, P wave maximum duration, and P wave terminal force with BMI 25–30 kg/m^2^ and BMI ≥ 30 kg/m^2^ compared to the reference group < 25 kg/m^2^ (*p* < 0.0001 in trends for all P wave indices).
	Liu, T., et al. [75]	Effect of obesity on P wave parameters in a Chinese population	Human	Standard 12-lead resting ECG. P(max) (111.9 +/− 9.3 vs. 101.1 +/− 6.0 ms, *p* < 0.01) and P wave duration and dispersion (P(d)) (47.9 +/− 9.3 vs. 31.8 +/− 6.9 ms, *p* < 0.01) were significantly prolonged in the obese group. P(min) was similar between the two groups. The prevalence of the inter-atrial block was significantly greater in the obese subjects. Pearson’s correlation analysis showed that there were positive correlations between P(d) and BMI (r = 0.6, *p* < 0.001), as well as between P(d) and the left atrial diameter (r = 0.366, *p* < 0.05)
	Babcock, M.J., et al. [76]	Pericardial fat and atrial conduction abnormalities in the Multiethnic Study of Atherosclerosis (MESA).	Human	Standard 12-lead resting ECG. All P wave indexes were significantly associated with pericardial fat (Pfat) in unadjusted analyses. After demographics adjustment, P-duration (1.68 (0.87, 2.49)) and P-terminal (0.16 (0.04, 0.28)), but not PR-duration (1.11 (−0.52, 2.74)) were associated with Pfat. No associations were significant after adjustment for BMI, waist circumference, or cardiovascular disease risk factors.
Voltage abnormalities	Mahajan, R., et al. [30]	Electroanatomical Remodeling of the Atria in Obesity: Impact of Adjacent Epicardial Fat	Human	Endocardial mapping (See above)
	Schram-Serban, C., et al. [38]	Low-voltage potentials contribute to postoperative atrial fibrillation development in obese patients	Human	Epicardial high resolution mapping. Compared with nonobese patients, obese patients have potentials with lower voltages (median of medians) (4.5 mV [0.4–16.2 mV] vs. 5.5 mV [0.8–18.0 mV]; *p* < 0.001), especially at Bachmann’s Bundle (4.1 mV [0.4–12.3 mV] vs. 6.2 mV [1.0–14.3 mV]; *p* < 0.001) and left atrium (5.1 mV [0.5–10.1 mV] vs. 6.2 mV [0.8–15.9 mV]; *p* = 0.003). The percentage of low-voltage potentials was higher in obese (median 3.6% [0.0–77.1%]) than in nonobese (median 2.3% [0.0–57.9%]) patients (*p* < 0.001).
	Klein, C., et al. [39]	Left atrial epicardial adipose tissue radiodensity is associated with electrophysiological properties of atrial myocardium in patients with atrial fibrillation	Human	Endocardial mapping. Patients with left atrial (LA) low-voltage zones (LVZ) presented significantly lower LA epicardial adipose tissue radiodensity than patients with no LA-LVZ (−101.8 ± 12.5 HU vs. −90.4 ± 6.3 HU, *p* = 0.004).
	Mahajan, R., et al. [29]	Electrophysiological, Electroanatomical, and Structural Remodeling of the Atria as Consequences of Sustained Obesity	Experimental (Ovis)	Endocardial mapping (See above)
	Lin, Y.K., et al. [36]	Adipocytes modulate the electrophysiology of atrial myocytes: implications in obesity-induced atrial fibrillation	Experimental (Leporidae)	Whole-cell patch clamp (See above)
	O’Connell, R.P., et al. [40]	Free Fatty Acid Effects on the Atrial Myocardium: Membrane Ionic Currents Are Remodeled by the Disruption of T-Tubular Architecture	Experimental (Ovis)	Isolated myocytes using the Langendorff retrograde perfusion method. Stearic acid disrupts t-tubular architecture and remodels properties of membrane ionic currents in sheep atrial myocytes.
Complex fractionated electrograms	Okumura, Y., et al. [28]	Effects of a high-fat diet on the electrical properties of porcine atria	Experimental (Suidae)	Endocardial mapping. Effective refractory periods of the pulmonary vein (PV) were shorter (*p* < 0.05), and AF lasted longer in the high fat diet group than in the control group (80 (45–1350) vs. 22 (3–30) s, *p* = 0.0212).
	Mahajan, R., et al. [30]	Electroanatomical Remodeling of the Atria in Obesity: Impact of Adjacent Epicardial Fat	Human	Endocardial mapping. (See above)
	Mahajan, R., et al. [29]	Electrophysiological, Electroanatomical, and Structural Remodeling of the Atria as Consequences of Sustained Obesity	Experimental (Ovis)	Endocardial mapping. (See above)
	Kanazawa, H., et al. [42]	Importance of pericardial fat in the formation of complex fractionated atrial electrogram region in atrial fibrillation	Human	Endocardial mapping. Total cardiac pericardial fat (PF) volume correlated with AF (odds ratio [OR]: 1.024, *p* < 0.001). Total cardiac PF volume and total CFAE area were both independently associated with persistence of AF (OR: 1.018, *p* = 0.018, OR: 1.144, *p* = 0.002, respectively). Multivariate linear regression analysis identified total cardiac PF volume as a significant and independent determinant of total CFAE area (r = 0.488, *p* < 0.001). Regional PF volume correlated with local CFAE area in each LA area.

## Data Availability

Not applicable.

## References

[1] Zhou M., Wang H., Chen J., Zhao L. (2020). Epicardial adipose tissue and atrial fibrillation: Possible mechanisms, potential therapies, and future directions. Pacing Clin. Electrophysiol..

[2] Wong C.X., Ganesan A.N., Selvanayagam J.B. (2017). Epicardial fat and atrial fibrillation: Current evidence, potential mechanisms, clinical implications, and future directions. Eur. Heart J..

[3] Ghattas K.N., Ilyas S., Al-Refai R., Maharjan R., Diaz Bustamante L., Khan S. (2020). Obesity and Atrial Fibrillation: Should We Screen for Atrial Fibrillation in Obese Individuals? A Comprehensive Review. Cureus.

[4] Goudis C.A., Korantzopoulos P., Ntalas I.V., Kallergis E.M., Ketikoglou D.G. (2015). Obesity and atrial fibrillation: A comprehensive review of the pathophysiological mechanisms and links. J. Cardiol..

[5] Menezes A.R., Lavie C.J., DiNicolantonio J.J., O’Keefe J., Morin D.P., Khatib S., Messerli F.H., Milani R.V. (2013). Cardiometabolic risk factors and atrial fibrillation. Rev. Cardiovasc. Med..

[6] Schram-Serban C., Heida A., Roos-Serote M.C., Knops P., Kik C., Brundel B., Bogers A.J., de Groot N.M. (2020). Heterogeneity in Conduction Underlies Obesity-Related Atrial Fibrillation Vulnerability. Circ. Arrhythm. Electrophysiol..

[7] Al-Rawahi M., Proietti R., Thanassoulis G. (2015). Pericardial fat and atrial fibrillation: Epidemiology, mechanisms and interventions. Int. J. Cardiol..

[8] Al Chekakie M.O., Welles C.C., Metoyer R., Ibrahim A., Shapira A.R., Cytron J., Santucci P., Wilber D.J., Akar J.G. (2010). Pericardial fat is independently associated with human atrial fibrillation. J. Am. Coll. Cardiol..

[9] Vroomen M., Olsthoorn J.R., Maesen B., L’espoir V., La Meir M., Das M., Maessen J.G., Crijns H.J.G.M., Verheule S., Pison L. (2019). Quantification of epicardial adipose tissue in patients undergoing hybrid ablation for atrial fibrillation. Eur. J. Cardiothorac. Surg..

[10] Shin S.Y., Yong H.S., Lim H.E., Na J.O., Choi C.U., Choi J.I., Kim S.H., Kim J.W., Kim E.J., Park S.W. (2011). Total and interatrial epicardial adipose tissues are independently associated with left atrial remodeling in patients with atrial fibrillation. J. Cardiovasc. Electrophysiol..

[11] Yorgun H., Canpolat U., Aytemir K., Hazırolan T., Şahiner L., Kaya E.B., Kabakci G., Tokgözoğlu L., Özer N., Oto A. (2015). Association of epicardial and peri-atrial adiposity with the presence and severity of non-valvular atrial fibrillation. Int. J. Cardiovasc. Imaging.

[12] Van Rosendael A.R., Dimitriu-Leen A.C., van Rosendael P.J., Leung M., Smit J.M., Saraste A., Knuuti J., van der Geest R.J., van der Arend B.W., van Zwet E.W. (2017). Association Between Posterior Left Atrial Adipose Tissue Mass and Atrial Fibrillation. Circ. Arrhythm. Electrophysiol..

[13] Mahabadi A.A., Lehmann N., Kälsch H., Bauer M., Dykun I., Kara K., Moebus S., Jöckel K.-H., Erbel R., Möhlenkamp S. (2014). Association of epicardial adipose tissue and left atrial size on non-contrast CT with atrial fibrillation: The Heinz Nixdorf Recall Study. Eur. Heart J. Cardiovasc. Imaging.

[14] Chu C.Y., Lee W.-H., Hsu P.-C., Lee M.-K., Lee H.-H., Chiu C.-A., Lin T.-H., Lee C.-S., Yen H.-W., Voon W.-C. (2016). Association of Increased Epicardial Adipose Tissue Thickness With Adverse Cardiovascular Outcomes in Patients with Atrial Fibrillation. Medicine.

[15] Hatem S.N., Redheuil A., Gandjbakhch E. (2016). Cardiac adipose tissue and atrial fibrillation: The perils of adiposity. Cardiovasc. Res..

[16] Hatem S.N., Sanders P. (2014). Epicardial adipose tissue and atrial fibrillation. Cardiovasc. Res..

[17] Batal O., Schoenhagen P., Shao M., Ayyad A.E., Van Wagoner D.R., Halliburton S.S., Tchou P.J., Chung M.K. (2010). Left atrial epicardial adiposity and atrial fibrillation. Circ. Arrhythm. Electrophysiol..

[18] Yamane T., Date T., Tokuda M., Aramaki Y., Inada K., Matsuo S., Shibayama K., Miyanaga S., Miyazaki H., Sugimoto K.-I. (2008). Hypoxemia in inferior pulmonary veins in supine position is dependent on obesity. Am. J. Respir. Crit. Care Med..

[19] Pathak R.K., Mahajan R., Lau D.H., Sanders P. (2015). The implications of obesity for cardiac arrhythmia mechanisms and management. Can. J. Cardiol..

[20] Haemers P., Hamdi H., Guedj K., Suffee N., Farahmand P., Popovic N., Claus P., LePrince P., Nicoletti A., Jalife J. (2017). Atrial fibrillation is associated with the fibrotic remodelling of adipose tissue in the subepicardium of human and sheep atria. Eur. Heart J..

[21] Burstein B., Nattel S. (2008). Atrial fibrosis: Mechanisms and clinical relevance in atrial fibrillation. J. Am. Coll. Cardiol..

[22] Greif M., von Ziegler F., Wakili R., Tittus J., Becker C., Helbig S., Laubender R.P., Schwarz W., D’anastasi M., Schenzle J. (2013). Increased pericardial adipose tissue is correlated with atrial fibrillation and left atrial dilatation. Clin. Res. Cardiol..

[23] Ayer J.G., Almafragy H.S., Patel A.A., Hellyer R.L., Celermajer D.S. (2008). Body mass index is an independent determinant of left atrial size. Heart Lung Circ..

[24] Ybarra J., Resmini E., Planas F., Navarro-López F., Webb S., Pou J.M., Santos A., Ballesta-López C. (2009). Relationship between adiponectin and left atrium size in uncomplicated obese patients: Adiponectin, a link between fat and heart. Obes. Surg..

[25] Sevinc D., Pasaoglu L., Coskun R., Atci N., Alimli A. (2016). Relationships between left atrial pericardial fat and permanent atrial fibrillation: Results of a case-control study. Diagn. Interv. Imaging.

[26] Gaborit B., Sengenes C., Ancel P., Jacquier A., Dutour A. (2017). Role of Epicardial Adipose Tissue in Health and Disease: A Matter of Fat?. Compr. Physiol..

[27] Hohl M., Lau D.H., Müller A., Elliott A.D., Linz B., Mahajan R., Hendriks J.M.L., Böhm M., Schotten U., Sanders P. (2017). Concomitant Obesity and Metabolic Syndrome Add to the Atrial Arrhythmogenic Phenotype in Male Hypertensive Rats. J. Am. Heart Assoc..

[28] Okumura Y., Watanabe I., Nagashima K., Sonoda K., Sasaki N., Kogawa R., Takahashi K., Iso K., Ohkubo K., Nakai T. (2015). Effects of a high-fat diet on the electrical properties of porcine atria. J. Arrhythm..

[29] Mahajan R., Nelson A., Pathak R.K., Middeldorp M.E., Wong C.X., Twomey D.J., Carbone A., Teo K., Agbaedeng T., Linz D. (2018). Electroanatomical Remodeling of the Atria in Obesity: Impact of Adjacent Epicardial Fat. JACC Clin. Electrophysiol..

[30] Mahajan R., Lau D.H., Brooks A.G., Shipp N.J., Manavis J., Wood J.P., Finnie J.W., Samuel C.S., Royce S.G., Twomey D.J. (2015). Electrophysiological, Electroanatomical, and Structural Remodeling of the Atria as Consequences of Sustained Obesity. J. Am. Coll. Cardiol..

[31] Munger T.M., Dong Y.X., Masaki M., Oh J.K., Mankad S.V., Borlaug B.A., Asirvatham S.J., Shen W.K., Lee H.C., Bielinski S.J. (2012). Electrophysiological and hemodynamic characteristics associated with obesity in patients with atrial fibrillation. J. Am. Coll. Cardiol..

[32] Nalliah C.J., Bell J.R., Raaijmakers A.J., Waddell H.M., Wells S.P., Bernasochi G.B., Montgomery M.K., Binny S., Watts T., Joshi S.B. (2020). Epicardial Adipose Tissue Accumulation Confers Atrial Conduction Abnormality. J. Am. Coll. Cardiol..

[33] Temiz F., Güneş H., Güneş H. (2019). Evaluation of Atrial Electromechanical Delay in Children with Obesity. Medicina.

[34] Vaidean G.D., Manczuk M., Magnani J.W. (2016). Atrial electrocardiography in obesity and hypertension: Clinical insights from the Polish-Norwegian Study (PONS). Obesity.

[35] Shirani J., Roberts W.C. (1993). Clinical, electrocardiographic and morphologic features of massive fatty deposits (“lipomatous hypertrophy”) in the atrial septum. J. Am. Coll. Cardiol..

[36] Lin Y.K., Chen Y.C., Chen J.H., Chen S.A., Chen Y.J. (2012). Adipocytes modulate the electrophysiology of atrial myocytes: Implications in obesity-induced atrial fibrillation. Basic Res. Cardiol..

[37] Takahashi K., Sasano T., Sugiyama K., Kurokawa J., Tamura N., Soejima Y., Sawabe M., Isobe M., Furukawa T. (2016). High-fat diet increases vulnerability to atrial arrhythmia by conduction disturbance via miR-27b. J. Mol. Cell Cardiol..

[38] Schram-Serban C., van Schie M.S., Knops P., Kik C., Bogers A.J., de Groot N.M. (2022). Low-voltage potentials contribute to postoperative atrial fibrillation development in obese patients. Heart Rhythm..

[39] Klein C., Brunereau J., Lacroix D., Ninni S., Brigadeau F., Klug D., Longere B., Montaigne D., Pontana F., Coisne A. (2019). Left atrial epicardial adipose tissue radiodensity is associated with electrophysiological properties of atrial myocardium in patients with atrial fibrillation. Eur. Radiol..

[40] O’connell R.P., Musa H., Gomez M.S.M., Avula U.M., Herron T.J., Kalifa J., Anumonwo J.M.B. (2015). Free Fatty Acid Effects on the Atrial Myocardium: Membrane Ionic Currents Are Remodeled by the Disruption of T-Tubular Architecture. PLoS ONE.

[41] Lioni L., Korantzopoulos P., Letsas K.P. (2011). Catheter Ablation of Atrial Fibrillation in Overweight and Obese Patients. J. Atr. Fibrillation.

[42] Kanazawa H., Yamabe H., Enomoto K., Koyama J., Morihisa K., Hoshiyama T., Matsui K., Ogawa H. (2014). Importance of pericardial fat in the formation of complex fractionated atrial electrogram region in atrial fibrillation. Int. J. Cardiol..

[43] Murthy S., Rizzi P., Mewton N., Strauss D.G., Liu C.Y., Volpe G.J., Marchlinski F.E., Spooner P., Berger R.D., Kellman P. (2014). Number of P-wave fragmentations on P-SAECG correlates with infiltrated atrial fat. Ann. Noninvasive Electrocardiol..

[44] Dublin S., French B., Glazer N.L., Wiggins K.L., Lumley T., Psaty B.M., Smith N.L., Heckbert S.R. (2006). Risk of new-onset atrial fibrillation in relation to body mass index. Arch. Intern. Med..

[45] Kusayama T., Furusho H., Kashiwagi H., Kato T., Murai H., Usui S., Kaneko S., Takamura M. (2016). Inflammation of left atrial epicardial adipose tissue is associated with paroxysmal atrial fibrillation. J. Cardiol..

[46] Lau D.H., Nattel S., Kalman J.M., Sanders P. (2017). Modifiable Risk Factors and Atrial Fibrillation. Circulation.

[47] Chalazan B., Dickerman D., Sridhar A., Farrell M., Gayle K., Samuels D.C., Shoemaker B., Darbar D. (2018). Relation of Body Mass Index to Symptom Burden in Patients withAtrial Fibrillation. Am. J. Cardiol..

[48] Abed H.S., Samuel C.S., Lau D.H., Kelly D.J., Royce S.G., Alasady M., Mahajan R., Kuklik P., Zhang Y., Brooks A.G. (2013). Obesity results in progressive atrial structural and electrical remodeling: Implications for atrial fibrillation. Heart Rhythm..

[49] Tsang T.S., Barnes M.E., Miyasaka Y., Cha S.S., Bailey K.R., Verzosa G.C., Seward J.B., Gersh B.J. (2008). Obesity as a risk factor for the progression of paroxysmal to permanent atrial fibrillation: A longitudinal cohort study of 21 years. Eur. Heart J..

[50] Zacharias A., Schwann T.A., Riordan C.J., Durham S.J., Shah A.S., Habib R.H. (2005). Obesity and risk of new-onset atrial fibrillation after cardiac surgery. Circulation.

[51] Wong C.X., Sullivan T., Sun M.T., Mahajan R., Pathak R.K., Middeldorp M., Twomey D., Ganesan A.N., Rangnekar G., Roberts-Thomson K.C. (2015). Obesity and the Risk of Incident, Post-Operative, and Post-Ablation Atrial Fibrillation: A Meta-Analysis of 626,603 Individuals in 51 Studies. JACC Clin. Electrophysiol..

[52] Kogo H., Sezai A., Osaka S., Shiono M., Tanaka M. (2019). Does Epicardial Adipose Tissue Influence Postoperative Atrial Fibrillation?. Ann. Thorac. Cardiovasc. Surg..

[53] Serban C., Arinze J.T., Starreveld R., Lanters E.A., Yaksh A., Kik C., Acardag Y., Knops P., Bogers A.J., de Groot N.M. (2020). The impact of obesity on early postoperative atrial fibrillation burden. J. Thorac. Cardiovasc. Surg..

[54] Guglin M., Maradia K., Chen R., Curtis A.B. (2011). Relation of obesity to recurrence rate and burden of atrial fibrillation. Am. J. Cardiol..

[55] Cha Y.M., Friedman P.A., Asirvatham S.J., Shen W.-K., Munger T.M., Rea R.F., Brady P.A., Jahangir A., Monahan K.H., Hodge D.O. (2008). Catheter ablation for atrial fibrillation in patients with obesity. Circulation.

[56] Chao T.F., Hung C.-L., Tsao H.-M., Lin Y.-J., Yun C.-H., Lai Y.-H., Chang S.-L., Lo L.-W., Hu Y.-F., Tuan T.-C. (2013). Epicardial adipose tissue thickness and ablation outcome of atrial fibrillation. PLoS ONE.

[57] Dereli S., Bayramoğlu A., Yontar O.C., Cerşit S., Gürsoy M.O. (2018). Epicardial fat thickness: A new predictor of successful electrical cardioversion and atrial fibrillation recurrence. Echocardiography.

[58] Okabe T., Buck B., Hayes S.A., Harfi T.T., Afzal M.R., Tyler J., Houmsse M., Kalbfleisch S.J., Weiss R., Hummel J.D. (2020). Extreme Obesity is Associated with Low Success Rate of Atrial Fibrillation Catheter Ablation. J. Atr. Fibrillation.

[59] Pathak R.K., Middeldorp M.E., Lau D.H., Mehta A.B., Mahajan R., Twomey D., Alasady M., Hanley L., Antic N.A., McEvoy R.D. (2014). Aggressive risk factor reduction study for atrial fibrillation and implications for the outcome of ablation: The ARREST-AF cohort study. J. Am. Coll. Cardiol..

[60] Glover B.M., Hong K.L., Dagres N., Arbelo E., Laroche C., Riahi S., Bertini M., Mikhaylov E.N., Galvin J., Kiliszek M. (2019). Impact of body mass index on the outcome of catheter ablation of atrial fibrillation. Heart.

[61] Letsas K.P., Siklódy C.H., Korantzopoulos P., Weber R., Bürkle G., Mihas C.C., Kalusche D., Arentz T. (2013). The impact of body mass index on the efficacy and safety of catheter ablation of atrial fibrillation. Int. J. Cardiol..

[62] Faroux L., Lesaffre F., Blanpain T., Mora C., Nazeyrollas P., Metz D. (2019). Impact of Obesity on Overall Radiation Exposure for Patients Who Underwent Radiofrequency Ablation of Atrial Fibrillation. Am. J. Cardiol..

[63] Mohanty S., Mohanty P., Di Biase L., Bai R., Dixon A., Burkhardt D., Gallinghouse J.G., Horton R., Sanchez J.E., Bailey S. (2011). Influence of body mass index on quality of life in atrial fibrillation patients undergoing catheter ablation. Heart Rhythm..

[64] Mohanty S., Mohanty P., Natale V., Trivedi C., Gianni C., Burkhardt J.D., Sanchez J.E., Horton R., Gallinghouse G.J., Hongo R. (2018). Impact of weight loss on ablation outcome in obese patients with longstanding persistent atrial fibrillation. J. Cardiovasc. Electrophysiol..

[65] Masuda M., Mizuno H., Enchi Y., Minamiguchi H., Konishi S., Ohtani T., Yamaguchi O., Okuyama Y., Nanto S., Sakata Y. (2015). Abundant epicardial adipose tissue surrounding the left atrium predicts early rather than late recurrence of atrial fibrillation after catheter ablation. J. Interv. Card. Electrophysiol..

[66] Ellis E.R., Reynolds M.R. (2012). Body Mass Index, Quality of Life, and Catheter Ablation in Patients with Atrial Fibrillation. J. Atr. Fibrillation.

[67] Mangiafico V., Saberwal B., Lavalle C., Raharja A., Ahmed Z., Papageorgiou N., Ahsan S. (2020). Impact of obesity on atrial fibrillation ablation. Arch. Cardiovasc. Dis..

[68] Guijian L., Jinchuan Y., Rongzeng D., Jun Q., Jun W., Wenqing Z. (2013). Impact of body mass index on atrial fibrillation recurrence: A meta-analysis of observational studies. Pacing Clin. Electrophysiol..

[69] Zhu W., Zhang H., Guo L., Hong K. (2016). Relationship between epicardial adipose tissue volume and atrial fibrillation: A systematic review and meta-analysis. Herz.

[70] Staerk L., Sherer J.A., Ko D., Benjamin E.J., Helm R.H. (2017). Atrial Fibrillation: Epidemiology, Pathophysiology, and Clinical Outcomes. Circ. Res..

[71] Abed H.S., Wittert G.A. (2013). Obesity and atrial fibrillation. Obes. Rev..

[72] Moher D., Liberati A., Tetzlaff J., Altman D.G., The PRISMA Group (2009). Preferred reporting items for systematic reviews and meta-analyses: The PRISMA statement. J. Clin. Epidemiol..

[73] Friedman D.J., Wang N., Meigs J.B., Hoffmann U., Massaro J.M., Fox C.S., Magnani J.W. (2014). Pericardial fat is associated with atrial conduction: The Framingham Heart Study. J. Am. Heart Assoc..

[74] Magnani J.W., Lopez F.L., Soliman E.Z., Maclehose R.F., Crow R.S., Alonso A. (2012). P wave indices, obesity, and the metabolic syndrome: The atherosclerosis risk in communities study. Obesity.

[75] Liu T., Fu Z., Korantzopoulos P., Zhang X., Wang S., Li G. (2010). Effect of obesity on p-wave parameters in a Chinese population. Ann. Noninvasive Electrocardiol..

[76] Babcock M.J., Soliman E.Z., Ding J., AKronmal R., Goff D.C. (2011). Pericardial fat and atrial conduction abnormalities in the Multiethnic Study of Atherosclerosis (MESA). Obesity.

[77] Sim I., Bishop M., O’neill M., Williams S.E. (2019). Left atrial voltage mapping: Defining and targeting the atrial fibrillation substrate. J. Interv. Card. Electrophysiol..

[78] Platonov P.G., Mitrofanova L.B., Orshanskaya V., Ho S.Y. (2011). Structural abnormalities in atrial walls are associated with presence and persistency of atrial fibrillation but not with age. J. Am. Coll. Cardiol..

[79] Latchamsetty R., Morady F. (2011). Complex fractionated atrial electrograms: A worthwhile target for ablation of atrial fibrillation?. Circ. Arrhythm. Electrophysiol..

[80] Esato M., Shimizu A., Chun Y.-H., Tatsuno H., Yamagata T., Matsuzaki M. (1996). Electrophysiologic effects of a class I antiarrhythmic agent, cibenzoline, on the refractoriness and conduction of the human atrium in vivo. J. Cardiovasc. Pharmacol..

[81] Wang T.J., Parise H., Levy D., D’Agostino R.B., Wolf P.A., Vasan R.S., Benjamin E.J. (2004). Obesity and the risk of new-onset atrial fibrillation. JAMA.

[82] Phan K., Khuong J.N., Xu J., Kanagaratnam A., Yan T.D. (2016). Obesity and postoperative atrial fibrillation in patients undergoing cardiac surgery: Systematic review and meta-analysis. Int. J. Cardiol..

[83] Csige I., Ujvárosy D., Szabó Z., Lőrincz I., Paragh G., Harangi M., Somodi S. (2018). The Impact of Obesity on the Cardiovascular System. J. Diabetes Res..

[84] Camm A.J., Kirchhof P., Lip G.Y., Schotten U., Savelieva I., Ernst S., Van Gelder I.C., Al-Attar N., European Heart Rhythm Association, European Association for Cardio-Thoracic Surgery (2010). Guidelines for the management of atrial fibrillation: The Task Force for the Management of Atrial Fibrillation of the European Society of Cardiology (ESC). Europace.

[85] Lau D.H., Schotten U., Mahajan R., Antic N.A., Hatem S.N., Pathak R.K., Hendriks J.M.L., Kalman J.M., Sanders P. (2016). Novel mechanisms in the pathogenesis of atrial fibrillation: Practical applications. Eur. Heart J..

[86] De Groot N.M.S., Shah D., Boyle P.M., Anter E., Clifford G.D., Deisenhofer I., Deneke T., van Dessel P., Doessel O., Dilaveris P. (2022). Critical appraisal of technologies to assess electrical activity during atrial fibrillation: A position paper from the European Heart Rhythm Association and European Society of Cardiology Working Group on eCardiology in collaboration with the Heart Rhythm Society, Asia Pacific Heart Rhythm Society, Latin American Heart Rhythm Society and Computing in Cardiology. Europace.

[87] Sun K., Halberg N., Khan M., Magalang U.J., Scherer P.E. (2013). Selective inhibition of hypoxia-inducible factor 1α ameliorates adipose tissue dysfunction. Mol. Cell Biol..

[88] Mahajan R., Lau D.H., Brooks A.G., Shipp N.J., Wood J.P.M., Manavis J., Samuel C.S., Patel K.P., Finnie J.W., Alasady M. (2021). Atrial Fibrillation and Obesity: Reverse Remodeling of Atrial Substrate With Weight Reduction. JACC Clin. Electrophysiol..

